# Investigation of the effect of Interleukin-1 receptor antagonist (IL-1ra) on markers of inflammation in non-ST elevation acute coronary syndromes (The MRC-ILA-HEART Study)

**DOI:** 10.1186/1745-6215-9-8

**Published:** 2008-02-25

**Authors:** David C Crossman, Allison C Morton, Julian P Gunn, John P Greenwood, Alistair S Hall, Keith AA Fox, Andrew J Lucking, Marcus D Flather, Belinda Lees, Claire E Foley

**Affiliations:** 1 Cardiovascular Research Unit, School of Medicine & Biomedical Sciences, University of Sheffield, Beech Hill Road, Sheffield, S10 2RX, UK; 2 Academic Unit of Cardiovascular Medicine, G Floor, Jubilee Wing, Leeds General Infirmary, Leeds, LS1 3EX, UK; 3 Centre for Cardiovascular Science, Queen's Medical Research Institute, University of Edinburgh, 47 Little France Crescent, Edinburgh, EH16 4TJ, UK; 4 Clinical Trials and Evaluation Unit, Royal Brompton and Harefield NHS Trust, Sydney Street, London, SW3 6NP, UK

## Abstract

**Background:**

Acute Coronary Syndromes account for 15% of deaths in the UK, and patients remain at significant risk of re-admission for future complications and death. Pathologically the underlying process of atherosclerosis is driven by inflammatory mechanisms, which are activated in ACS patients. Previous studies have investigated the role of inflammatory markers in this process, including interleukin 1 (IL-1) and C Reactive Protein (CRP). Pre-clinical studies indicate that IL-1 may be a primary driver of ACS and that the naturally occurring interleukin-1 receptor antagonist (IL-1ra) may inhibit the atherosclerotic process. This study will investigate the effects of IL-1ra on inflammatory markers in man.

**Methods/design:**

Three centres in the UK are planning to recruit 186 Non-ST elevation myocardial infarction patients to receive either interleukin-1 receptor antagonist (Anakinra) or matching placebo. Patients will receive a daily subcutaneous injection of either study drug or placebo over a 14 day period. The primary outcome is area under the curve of high sensitivity C-Reactive Protein (CRP) over the first 7 days.

**Discussion:**

The MRC-ILA-HEART Study is a proof of concept clinical trial investigating the effects of IL-1ra upon markers of inflammation in patients with Non-ST elevation myocardial infarction. It is hoped this will provide new and exciting information in relation to an "anti-inflammatory" strategy for patients with acute coronary syndrome.

**Trial registration:**

ISRCTN89369318

## Background

Acute coronary syndromes (ACS), caused by coronary atherosclerotic plaque destabilisation and interruption of coronary flow, are common, serious conditions that account for 15% of all deaths in the UK and are associated with considerable morbidity among survivors. ACS are classified by the presenting ECG: patients with persistent ST elevation myocardial infarction (STEMI) generally require early reperfusion [1], whereas those with non ST-elevation (NSTEMI) ACS require early risk stratification and revascularisation as necessary [2]. There remains, however, considerable risk in the non-ST elevation ACS patients: the average risk of death at 6 months is 6.2% with 19.3% re-attending hospital because of heart disease [3]. It is known that inflammatory processes are activated in ACS. This proposal is for a proof of concept clinical trial of a new and exciting anti-inflammatory strategy for the treatment of NSTEMI ACS based on pre-clinical investigations that we wish to translate to man.

### Inflammation and atherosclerosis

The past decade has seen a growth in treatment options available for ACS as a consequence of better understanding of the pathophysiology of this condition. At a biological level the process is driven by inflammatory mechanisms in the vessel wall where atherogenesis is initiated by lipids and other environmental agents [4,5]. Instability of the plaque atheroma so as to cause the disease to present is also viewed as an inflammatory process, predominantly where plaque macrophages destabilise the cap of the atheroma, resulting in the formation of an intra-coronary thrombus.

The therapeutic implications of these pathogenic pathways are that primary and, to some extent, secondary prevention are targeted at mutable risk factors (smoking cessation, blood pressure control, lipid lowering etc.) and anti-thrombotic strategies for the treatment and prevention of intracoronary thrombus. Evaluation of therapeutic strategies designed to treat the inflammatory processes involved in acute or chronic coronary artery disease (CAD) is at an early stage. Some agents may potentially have anti-inflammatory properties (statins in particular and fish oils) but this is not their primary mechanism of action and proving beneficial effects consequent upon the anti-inflammatory properties of these agents has been difficult.

Since the description of ACS as an inflammatory event potential sources of, and functions for, inflammatory cytokines in this process have been investigated [5,6]. IL-1 is an apical cytokine in the innate inflammatory response transducing signals from bacterial products through pattern recognition receptors, complement activation and TNFα. IL-1 has plausible actions in arterial wall inflammation in the setting of atherosclerosis, in particular its effects on endothelial cells (activation) and vascular smooth muscle cells (proliferation). There are two agonistic cytokines (IL-1α and IL-1β) that signal via the Type 1 IL-1 receptor and there is a specific Interleukin-1 receptor antagonist (IL-1ra), the only known naturally occurring cytokine antagonist, that inhibits IL-1 actions by binding to the Type 1 IL-1 Receptor (IL-1RI), blocking the receptor and preventing IL-1 from binding thereby preventing receptor signalling [7].

### Relevance of interleukin-1

The suggestion that IL-1 is involved in the pathogenesis of CAD, and in particular in ACS, is derived from a number of sources as there is no animal model of this condition. IL-1 and IL-1ra are expressed in diseased human coronary arteries [8,9]. Coronary artery IL-1β is up-regulated in porcine coronary arteries after balloon injury [10]. Genetic studies have indicated that polymorphic variants within the IL-1 cluster that have net pro-inflammatory effects are associated with angiographic CAD [11,12].

In mouse models of atherosclerosis IL-1ra inhibits the formation of intimal fatty streaks in apolipoprotein E-deficient mice [13], and IL-1β deficient mice when crossed onto an apo e-/- background have reduced atherosclerosis [14]. Relevant to vessel wall healing, we have data from mice and pigs that indicate the vessel wall response to injury (neointima formation) is an intensely IL-1 dependent event [15]. In the porcine coronary artery, the neointima response to balloon angioplasty (a model of arterial wall rupture in some way analogous to that which occurs in ACS) is reduced in its amount by a 14 day s.c. infusion of IL-1ra. We have crossed the IL-1R1-/- mouse with the apo e-/- and found reduced atherosclerosis. This double knockout, unable to signal IL-1, has no rise in blood pressure with fat feeding (in contrast to the apo e-/- control mice) and this is associated with reduced serum amyloid A levels (mice do not appear to have CRP), reduced markers of oxidative stress in the arterial wall and preservation of endothelial cell function in resistance arteries. We have reproduced the blood pressure rise inhibition and the modulation of oxidative stress seen in the IL-1R1-/-, apo e-/- double knockout by using s.c. IL-1ra treatment in apo e-/- mice.

In a cohort of 63 NSTEMI ACS patients we examined the impact of IL-1 gene polymorphisms upon markers of endothelial cell activation and CRP [16]. The intron 2, VNTR polymorphism that we have previously described as associated with reduced endothelial cell production of IL-1ra [17] (i.e. endothelial pro-inflammatory) was associated with significant increases in soluble markers of endothelial activation at the time of NSTEMI ACS, including the rise in plasma vWF, a marker known to be associated with clinical outcome [18].

Overall, our preliminary data indicate that 1) IL-1 is a potent mediator of arterial inflammation in the context of atherosclerosis and vessel wall injury and 2) IL-1 inhibition restores endothelial function, reduces inflammatory vessel wall responses and reduces vascular oxidative stress. We wish now to translate these findings to man.

### Markers of inflammation in ACS

ACS, as has been stated before, is an intensely inflammatory event at the level of the vessel wall. Data from angioscopy [19], angiography [20], IVUS [21] as well as functional cell biology [22] indicate that ACS is frequently associated with widespread coronary activation and inflammation, indicating a pancoronary inflammatory state. It has also become clear that serum markers of inflammation are predictors of complications following an episode of ACS and that statin therapy associated with lower achieved levels of CRP are associated with improvement in outcome [23]. These data suggest that modulation of inflammation in the coronary artery would be predicted to translate to clinical benefit. There is considerable interest in markers of outcome in ACS. These broadly fall into two groups; those that measure the size of the infarct and those that are measures of the pathogenic process in the vessel way and may reflect both the magnitude and duration of the process. In the former group are the markers of myocyte damage. Cardiac-specific Troponin is now recognised as a more sensitive and specific marker of myocardial necrosis than creatine kinase and its MB isoenzyme. Elevation of serum Troponin has been shown to identify patients with ACS at risk for adverse clinical outcomes [24-26].

In the latter group (markers of the pathological process) are the markers of inflammation, coagulation and of platelet activation. We believe that there are plausible links between IL-1 activation and these markers. The acute phase reactant C-reactive protein (CRP) is a strong predictor of cardiovascular events [27,28]. In NSTEMI ACS, treated with percutaneous coronary intervention (PCI), CRP rises and peaks between 24 and 48 hours [29]. It is clear, however, that in some patients there is persistent elevation with maybe 49% of patients having a CRP >3 mg/L at discharge and these patients were identified as having increased chances of recurrent admissions [30]. The absolute levels of CRP have been shown to have a high sensitivity (87%) and a low specificity (54%) for subsequent myocardial infarction in patients presenting with an ACS [31]. There is a difficulty using a percentage change in CRP, either for individual patients and predicting outcome or between two groups of (treated) patients, in that there is no baseline level from which comparisons can be made. Differences in outcomes in the PROVE-IT trial, however, were associated with differences in the achieved CRP level [23,32]. Biologically, CRP production by hepatocytes has to be induced and this has been shown to be driven by the IL-1 and IL-6 cytokines. Serum levels of IL-6 have been shown to be elevated in patients with ACS [33] and an elevation of IL-6 is an independent marker of increased mortality in ACS [30]. The relationship between IL-1 and IL-6 is well defined with IL-1 inducing IL-6 production [7]. It has, however, proved difficult to reliably measure IL-1β; a cytokine that is devoid of a signal sequence directing release and which probably circulates at a very low level.

During NSTEMI, a rise in von Willebrand Factor (vWF) between 24 and 48 h predicts adverse clinical events [18], and an association between circulating levels of IL-6 and von Willebrand factor (vWF) has recently been shown to determine prognosis in patients with ACS [34]. IL-1 has been shown to augment thrombin-induced release of vWF from human endothelial cells [35].

### Interleukin 1 receptor antagonist (IL-1ra)

There are two agonistic cytokines (IL-1α and IL-1β) that signal via the Type 1 IL-1 receptor (IL-1R1) and a specific Interleukin-1 receptor antagonist (IL-1ra), (the only known naturally occurring cytokine antagonist) that inhibits IL-1 actions by binding to the IL-1R1, blocking the receptor and preventing IL-1 from binding thereby preventing receptor signalling [7].

IL-1ra is available for the treatment of a number of inflammatory conditions as a recombinant, non-glycosylated, N-terminal methionine added protein (Kineret/Anakinra, Amgen). Although less successful than anti-TNF therapies in rheumatoid arthritis (RA), there appears to be considerable benefit in the management of Juvenile arthritis (Still's disease), and it is the treatment of choice in the rare autoinflammatory illnesses typified by Muckle-Wells syndrome. From all of the indications in which IL-1ra has been used it has emerged that IL-1ra is a very safe form of treatment and appears to be devoid of the side-effect of re-activation of infectious illness seen with anti-TNF therapies. The only problems encountered are mild, reversible injection site reactions and neutropaenia in <1% of patients after a month or more of treatment.

Dosing with IL-1ra is simple. A single, daily, subcutaneous 100 mg injection of IL-1ra is the standard dose of IL-1ra for all individuals with normal renal function. In RA this causes a 35% reduction in CRP after approximately 1 week of treatment. Higher doses do not achieve any greater reduction in CRP of relief of RA symptoms. Single daily dosing schedules are used in the sucessful treatment of Juvenile arthritis and Muckle-Wells. The half-life of IL-1ra is 4–6 hours with maximum absorption from an s.c. injection at 3–7 hours. (Data on file supplied by Amgen).

## Conclusion

All of these data indicate that the IL-1 cytokine has the potential to act as a primary driver of ACS. Prolonged coronary activation is associated with worse outcome in ACS. It is therefore plausible that blockade of the IL-1 system could be of therapeutic benefit. We believe our pre-clinical data indicates that a purely anti-inflammatory approach based upon Interleukin-1 (IL-1) inhibition using IL-1 receptor antagonist (IL-1ra) is a feasible and logical therapy for ACS and this approach could open a new therapeutic paradigm for evaluation in further studies.

### Aim of study

The main objective is to assess whether treatment of NSTEMI ACS with IL-1ra alters the inflammatory process involved in this condition

### Trial design

Randomised, double blind, placebo-controlled multi-centre phase II clinical trial

### Eligibility

#### Inclusion criteria

1. Acute cardiac chest pain consistent with an acute coronary syndrome

2. <48 hours from onset of symptoms that led to hospital admission

3. And at least one of the following:

a) A raised troponin as defined by local parameters specified at each centre

b) Other ECG changes consistent with acute myocardial ischaemia (e.g. Horizontal or down-sloping ST depression of at least 0.5 mm or T-wave inversion of at least 3 mm, in at least two leads of the ECG, or new onset bundle branch block) and an elevated level of Troponin above local laboratory values indicating myocardial damage

#### Exclusion criteria

1. < 18 years of age

2. Persistent ST elevation on the presenting ECG

3. Intention to treat with an urgent reperfusion strategy (thrombolysis or primary percutaneous coronary intervention)Percutaneous coronary intervention within previous 3 months

4. Previous coronary artery bypass grafting

5. ECG showing paced rhythm

6. Cardiogenic shock (as defined in the Trial Manual)

7. Any serious co-morbidity which makes it unlikely that the patient will complete trial procedures and follow-up

8. Treatment or under active follow-up for rheumatoid arthritis, other connective tissue diseases or inflammatory bowel disease

9. End stage renal disease or a Creatinine > 220 μmol/L

10. Pregnancy or suspected pregnancy (any potential female participant of child bearing age will need a negative pregnancy test prior to study entry)

11. Eosinophilia

12. Anti-TNF biologies

13. Active infection

14. Malignancy

### Study treatment

Eligible patients will be randomised in equal proportions between IL-1ra and placebo, receiving either a once daily, subcutaneous (s.c). injection of IL-1ra (dose 100 mg per 24 h) for 14 days, or a daily s.c. injection of placebo for 14 days. The hospital pharmacist will issue the relevant drug package. (Please see appendices for pharmacy information).

The study drug and placebo will be provided by Amgen Inc in its commercially available recombinant form which is in clinical use for the treatment of arthritic conditions. The study drug and placebo will be relabelled by Amgen, in collaboration with CTEU according to MHRA guidelines.

The first dose of IL-1ra will be given within 24 h +2 h of the positive Troponin. Injections will be given at a standardised time (24 ± 2 h after the previous dose), immediately after blood sampling. IL-1ra or placebo will administered to the patient by the research nurse while the patient is in hospital. During the hospital stay, the patient will be taught to self-administer the injection by the research nurse and on discharge will continue at home. This has proven possible in other ACS trials that required self injection of subcutaneous heparin [36]. Full written guidance on self injection will also be provided to patients. If self injection is found not to be possible in an individual patient for unexpected reasons, an alternative method will be sought (e.g. district nurse, or attending the hospital) to try and maintain full compliance with scheduled study drug regimen after discharge. Patients will also be asked to complete a daily injection diary. All personnel will be blinded to the identity of the syringe contents.

### Concomitant treatments and procedures

All patients will receive standardised additional treatments according to best practice including aspirin, clopidogrel, heparin (intravenous or low-molecular weight), a glycoprotein IIb/IIIa inhibitor and a statin. Other medication will be at the physicians' discretion. It is the intention that all patients will proceed to coronary angiography according to current guidelines, and subsequent revascularisation if necessary.

### Drug composition

Each vial contains 100 mg of anakinra in 1 ml (100 mg/ml). Anakinra is a human interleukin – 1 receptor antagonist (r -metHuIL -1ra) produced by recombinant DNA technology in an E coli expression system.

### Marketing authorisation Holder

Amgen Europe B.V., Minervum 7061, 4817 ZK Breda, The Netherlands

### Placebo composition

Each vial consists of sodium citrate, sodium chloride, disodium edentate, polysorbate 80, sodium hydroxide and water for injection.

### Screening and enrolment

Participating centres and investigators have extensive experience of clinical trials and admit large numbers of patients with acute coronary syndromes. It is expected that screening for eligible patients will occur on a daily basis and potentially eligible patients will be approached for consent to participate. Screening for patients should occur routinely in ward areas that admit ACS patients. Centres are encouraged to screen and enrol at the weekends and out of hours depending on local resources. Each centre is requested to randomise and follow-up an average of 62 patients (30 per annum or 2–3 patients per month) over a 2 year period.

### Screening procedure

A member of the research team will screen patients for eligibility as soon as possible after presentation. If patients meet the eligibility criteria they will be given the patient information sheet to consider. Patients will be given as much time as they require to consider taking part but due to the acute nature of this condition, patients will be recruited as soon as possible after the positive troponin is confirmed. A TIMI (Thrombolysis in Myocardial Infarction) and GRACE risk score will be determined for each patient at baseline [1,37].

### Informed consent

"Informed consent" requires individual discussion with the patient about the nature of the procedures to be conducted in a language that is easy to comprehend. The patient should fully understand that he/she might be allocated to either the IL-1ra or the placebo group and the potential adverse effects of participation. The patient should also understand that his/her refusal to participate in the study will not affect the quality of subsequent medical care and if they do consent to participate they may withdraw at any point without affecting their care.

Before any trial-related procedures may be performed, informed consent must be obtained from the patient by the investigator by means of a signed declaration. The investigator must sign in the CRF to confirm that informed consent was obtained and store the original of the signed declaration of consent in the patient's notes. A copy should be given to the patient and a copy filed in the CRF.

### Randomisation

Randomisation will be performed using a 24-hour voice interactive system set up the trial co-ordinating centre (Clinical Trials and Evaluation Unit (CTEU) of the Royal Brompton Hospital, London). Centres will be asked for a few simple details about the patient including initials, date of birth and eligibility criteria. The caller will be given a randomisation number, corresponding to blinded study treatment, and a fax or e mail will be sent to the centre confirming this. Randomisation will be stratified by centre and by whether glycoprotein IIb/IIIa antagonists are being used or planned. Patients entered into the study in this way will be considered as part of the intention to treat cohort irrespective of whether they actually receive treatment or not, or if they subsequently with draw from the study.

### Study related investigations and follow-up

Patients will have blood taken every day for seven days for hsCRP (baseline and days 1–7, 14 and 30), Troponin (baseline and days 1–7 and 14), full blood count (baseline, days 7 and 14), creatinine, electrolytes, liver function tests (baseline, days 1–7 and 14), Interleukin-6 and von Willebrand factor (baseline, days 1–3,14,30). Blood will be taken prior to administration of either IL-1ra or placebo. A summary of samples and study related procedures to be undertaken is shown in Table 1. If the patient is discharged before the seventh day, they will be required to attend clinic to have the remaining blood samples taken.

**Table 1:** Study procedures

Procedure	Baseline	Days 1 – 7	Days 8–13	Day 14	Day 30 (end of study)
Clinical evaluation	√	√		√	√
Informed consent	√				
Randomisation	√				
ECG	√	√		√	
Study drug given		√	√	√	
Full blood count	√	√		√	
Creatinine, electrolytes, eGFR	√	√		√	
Troponin	√	√		√	
hsCRP	√	√		√	√
Liver function tests	√			√	
IL-6, vWF	√	√		√	√
Post discharge follow up visit				√	√
Samples for DNA & RNA	√				√
Cardiac magnetic resonance scan (1)		√			√
ST segment monitoring (2)		√			
Forearm endothelial study (3)		√		√	

### Participant follow up

All patients will be followed up for MACE at 30 days (at clinic), three months and one year by telephone call with the Research Nurse. Patients will also be followed up for up to five years by the ONS.

### Collection of blood for DNA/RNA

Common genetic variations in the IL-1β gene have been shown to influence IL-1 β production and been implicated in the causation of premature coronary artery disease. IL-1β effects are mediated via the IL-1 receptor that is antagonised by IL-1ra. As with other receptors, stimulation may result in down-regulation and inhibition in up-regulation. We aim to demonstrate this by measurement of IL-1r mRNA expression. Genomic DNA and mRNA samples will be collected, stored and studied. These will be used to study the genetic substrate of observed differences in inflammatory markers and also effects of IL-1ra treatment – with initial experimental questions being as outlined here. Two additional blood samples will be taken at base-line and follow up at day 30 (1) 6 ml blood in EDTA tube for DNA extraction using the 'Versagene' DNA purification system (2) 5 ml blood in 'PAXgene' vacuum tube.

### Sub-studies

There are three sub studies proposed for the study (please see appendix 1.2. for protocols) including:

1. Cardiac Magnetic Resonance (CMR) – Leeds Patients only

2. ST-Holter segment analysis – Sheffield patients only

3. Forearm Endothelial Cell response – Edinburgh patients only

### Outcome measures

#### Primary outcome measure (comparison of treatment group with control)

Area under the curve of serum high sensitivity CRP over the first 7 days

#### Secondary outcome measures (comparison between treatment and control of)

1. Mean hsCRP at 7, 14 and 30 days

2. Area under the curve of Troponin-I

3. vWF and IL-6

4. ST segment depression on Holter monitor

5. Myocardial injury as determined by Gadolinium enhanced CMR scan

6. Forearm endothelial cell response

7. Incidence of major adverse cardiovascular events (MACE) at 30-days, three months and at one year.

8. Flagging with Office of National Statistics (ONS) for up to 5 years.

### Statistics

#### Sample size

To provide the study with an 80% power to detect a relative reduction of one-third (the reduction seen in IL-1ra treatment of rheumatoid arthritis at this dose (data on file [Amgen]) between the treatment and the placebo groups at a 5% level of significance will require approximately 80 patients in each group (total 160). This is based upon the log-transformed value of the area under the curve and a standardised difference (difference/standard deviation [D]) of approximately 0.45. The standardised difference had been used since it is very difficult to estimate what the values of the log-transformed AUC will be. A change of 0.45 on the standardised normal will correspond to a one-third change in the primary outcome, regardless of the scale of the measurement, assuming the outcome measure is normally distributed. As the sample size is sufficiently powered to detect a change equivalent to one-third on the standard normal, it will also be sufficient to detect the same level of change on the primary outcome measure. It is anticipated that follow-up will be near complete in this study. However, allowing for data loss, for whatever reason, of 10% in each arm we have increased the required sample size to approximately 180 patients (60/centre). Furthermore, the sample size needs to be adjusted for the 2 interim analyses that will be conducted during the trial. Using the method of O'Brien and Flemming (1979) gives an increased sample size of 184 patients.).

#### Interim analyses

A limited number of interim analyses will be performed by the trial statistician as specified by the Data Monitoring Committee (DMC). The accumulating results will not be available to the trialists or other principal investigators. The final, definitive analysis of the trial data will be conducted 25 months after the date of commencement of the trial. The interim analysis will test for efficacy, safety and futility. The method of O'Brien and Fleming suggests that stopping for efficacy will only occur if the first interim analysis shows a treatment effect equivalent to p < 0.0005 or if the second interim analysis shows a treatment effect equivalent to p < 0.014.

#### Primary analysis

Primary analysis will be of log-transformed AUC of 7 day CRP. The primary analysis will include all patients with at least 4 follow-up readings, on an intention-to-treat basis, and a secondary analysis will include only those with all follow-up readings on a per-protocol basis. ANOVA (analysis of variance) will be used to analyse the primary outcome measure and to estimate the treatment effect. For patients with at least 4 readings, any missing measurements will be imputed using the last observation carried forward method. After adjustment for the interim analyses, based on the method of O'Brien and Fleming, the primary endpoint will be considered to be statistically significant if it shows a treatment effect equivalent to p < 0.045.

### Trial organisation and committees

#### Trial steering committee (TSC)

The main role of the TSC is to monitor and supervise the progress of the trial. The TSC membership is listed in Table 2.

**Table 2:** Trial steering committee membership

Name	Trial Role	Title	Affiliation
Professor D Crossman	Chief Investigator	Professor of Cardiology	Sheffield
Dr Philippa Tyrrell	Independent Chair	Senior Lecturer in Stroke	Manchester
Dr Mark DeBelder	Independent Cardiologist	Consultant Cardiologist	South Tees
Dr A Morton	Co-investigator	Clinical Lecturer in Cardiology	Sheffield
Professor A Hall	Co-investigator	Professor of Cardiology	Leeds
Professor K Fox	Co-investigator	Professor of Cardiology	Edinburgh
Mr Michael Roughton	Study Statistician	Statistician, Royal Brompton Hospital	London
Dr M Flather	Co-investigator	Director, CTEU, Royal Brompton Hospital	London
Dr J Greenwood	Co-investigator	Senior Lecturer in Cardiology	Leeds
Dr J Gunn	Co-investigator	Senior Lecturer in Cardiology	Sheffield
Mrs Bev Kilner	Recruiting Nurse	Research Sister	Sheffield
Mr Graham Seaton	Lay member		

Representatives from the sponsor organisation, University of Sheffield, and funding organisation, Medical Research Council, will attend the Trial Steering Committee as observers.

#### Data monitoring committee (DMC)

The main role of the DMC is to consider the data from any interim analyses and specifically to assess any safety issues (such as unexpected serious adverse events) that occur and report back to the TSC. The DMC membership is listed in Table 3. All members of the DMC are independent of the trial. The DMC will meet prior to the start of the trial and then one and two thirds of the way through the Trial or as required thereafter. The DMC will be expected to develop, in agreement with the investigators, a charter outlining their responsibilities and operational details.

**Table 3:** Data monitoring committee membership

Name	Trial Role	Title	Affiliation
Professor R Willcox	Chair	Professor of Cardiology	Nottingham
Dr Allan Skene	Statistician	Managing Director of Nottingham Clinical Research Group	Nottingham
Dr E Grech	Cardiology advisor	Consultant Cardiologist	Sheffield

#### Clinical event review committee

The Clinical Event Review Committee will review adverse events during the study and adjudicate them to ensure the events meet the definitions given. Each event will be independently adjudicated by two committee members. A third committee member will be called to adjudicate an event if agreement is not reached by two members. The adjudications will be blind to the knowledge of which arm of the trial the patients are in.

#### Study co-ordination

The study will be co-ordinated and managed by the Clinical Trials and Evaluation Unit (CTEU) a dedicated clinical trials department within the Royal Brompton Hospital. In addition to providing overall project co-ordination, the CTEU will assist in preparing the final protocol, the investigators' manuals, design the Case Report Forms (CRF), provide the randomisation service and design and instigate the data management system. The CTEU will ensure that the trial runs according to the pre-agreed timetable, recruitment targets are met, CRFs are completed accurately, compliance with relevant ethical and regulatory standards, and that all aspects of the study are performed to the highest quality. The CTEU will also assist in the training of investigators and co-ordinators at the start-up of the study and for performing monitoring procedures throughout. A smaller trial management team consisting of lead investigators at each site and CTEU will meet weekly by conference call.

### Regulatory issues

#### Study sites

Support for patient identification, study administration, data collection and follow-up will be provided to each participating site. Study site co-ordinators will be responsible for screening patients (and recording the data on a screening log), enrolling patients into the trial, providing a contact point for patients, liaising with CTEU, completing CRFs, arranging all follow up visits and measurements, recording adverse events, ensuring forms are sent to CTEU and that all edit queries are resolved.

#### Data collection

Each centre will be provided with a Protocol, Manual of Operations, questionnaires and patient CRFs. Data will be recorded onto two part NCR CRFs and the top copy sent to the CTEU at the times specified.

#### Investigators' responsibilities

Investigators must ensure that Local Ethics Committee approval has been obtained as well as Agreements signed off by their Institution prior to the start of the study. Investigators are responsible for performing the study in accordance with the European Clinical Trials Directive, the Medicines for Human Use (Clinical Trials) Regulations 2004, NHS Research Governance, MRC Guidelines for Good Clinical Practice in Clinical Trials (1998) and the Declaration of Helsinki guidelines. Investigators are required to ensure compliance to the protocol, CRFs and Manual of Operations. Investigators are required to allow access to study documentation or source data on request for monitoring visits and audits performed by the CTEU or any regulatory authorities.

#### Pre-study training visit

Before the study commences each centre will receive a training visit by CTEU. These visits will ensure that personnel at each site (including principal investigators, co-investigators, study site co-ordinator and pharmacists) fully understand the protocol, CRF and the practical procedures for the study.

#### Monitoring visits

At regular intervals during the study CTEU will perform monitoring visits to each centre. The purpose of these visits is to ensure compliance to the protocol and that ethical and regulatory guidelines are met. Source data verification and checking of essential documents will be performed. Monitoring visits also provide an opportunity for further training if required (eg new staff). Central review of study data will also be performed throughout the study.

#### Close out visit

At the end of the study each centre will receive a site visit from CTEU to resolve any outstanding edit queries or adverse events and to verify the correct storage of study documentation.

#### Regulatory framework and approval

This study is a randomised trial of a medicinal product in a new indication. The IL1-receptor antagonist is commercially available and approved for use in specific inflammatory conditions. As such the study will need to comply with the European Clinical Trials Directive and the Medicines for Human Use (Clinical Trials) Regulations 2004. An application will be made for a clinical trials authorisation (CTA) to the Medicines and Healthcare Regulatory Agency (MHRA) prior to starting the study. The study has been registered in the European Community with a Eudract number.

#### Study sponsor

The grant has been awarded to the University of Sheffield who will act as the main Sponsor of the study. The Sponsor's role is clearly set out in the European Clinical Trials Directive and NHS Research Governance documents. Research agreements will be held with the 4 collaborating groups: University of Edinburgh/Royal Infirmary of Edinburgh, University Hospitals of Leeds, Sheffield Hospitals NHS Trust and Royal Brompton and Harefield NHS Trust. The Clinical Trials and Evaluation Unit of the Royal Brompton will be responsible, on behalf of the University of Sheffield, for ensuring that regulatory compliance is carried according to the high standards expected.

#### Safety reporting

The University of Sheffield (Sponsor) has delegated responsibility for pharmacovigilance to the trial co-ordinating centre (Clinical Trials and Evaluation Unit (CTEU) of the Royal Brompton Hospital, London).

The CTEU will be responsible for recording all reported serious adverse events from investigational trial sites, reporting all serious adverse events to a constituted serious adverse events committee (SAEC) for review and expedited reporting of suspected unexpected serious adverse reactions (SUSARs) in accordance with statutory regulations. The SAEC will consist of one nominated investigator from each trial site who can provide clinical expertise to adjudicate serious adverse events and determine if they are SUSARs, and advise the CTEU whether expedited reporting is required.

### Adverse reactions (AR)

An adverse reaction of an investigational medicinal product (AR) can be defined as all untoward and unintended responses to an investigational medicinal product related to any dose administered.

In the event an AR is reported during the trial, investigators will assess the severity of the adverse event using the following criteria, detailed on the adverse event report form in the case report form (CRF):

Mild: Awareness of signs or symptoms, but easily tolerated; are of minor irritant type; causing no loss of time from normal activities; symptoms would not require medication.

Moderate: Discomfort severe enough to cause interference with usual activities.

Severe: Inability to do work or usual activities; signs and symptoms may be of systemic nature or require medical evaluation and/or treatment.

#### Drug related adverse reactions

In all placebo-controlled trials conducted by Amgen, injection site reactions (ISRs) were frequently reported in the majority of patients (95% of which were reported to be mild to moderate in severity). ISRs included erythema, ecchymosis, inflammation and pain. On a dose of 100 mg/day, 71% of patients developed an ISR (n = 1565) compared to 28% of placebo treated patients (n = 733). The median duration of typical symptoms was 14 to 28 days. Other adverse events reported by patients administered with IL-1ra were, URI, headache, nausea, diarrhoea, sinusitis, arthralgia, flu like symptoms and abdominal pain. Allergic reactions reported in patients were rare.

Reported ISRs and other drug related adverse reactions will be reported on the adverse event report in the CRF. Patients reporting ISRs will be required to identify another injection site in which to administer the injection. Investigators will submit all adverse events reports for each patient to the CTEU at days 7, 14 and 30, three months and one year. The CTEU will maintain a log of all adverse reactions.

#### Serious adverse events/reactions

Serious adverse events (SAE) or reactions can be defined as any untoward medical occurrence or effect that at any dose

• results in death,

• is life-threatening,

• requires hospitalisation or prolongation of existing inpatients hospitalisation,

• results in persistent or significant disability or incapacity,

• is a congenital anomaly or birth defect.

#### Expected serious adverse events/clinical outcomes due to underlying acute coronary syndrome

• Myocardial Infarction (MI)

• Stroke

• Revascularisation

• Major Bleed

• Death

#### Drug related serious adverse reactions

• Serious Infections – Previous trials conducted by Amgen demonstrated an incidence of serious infections in 1.8% of IL-1ra treated patients compared to 0.7% in placebo group. There were no on-study deaths due to serious infectious episodes.

• Neutropenia – Administration of IL-1ra was associated with neutropenia (ANC < 1.5 × 10^9^/I) in 2.4% of patients compared with 0.4% of placebo patients. None of these patients had serious infections associated with the neutropenia.

#### Reporting of serious adverse events/reactions

The CRF will contain specific SAE reports relating to the SAEs/clinical outcomes listed above. The investigator will complete the SAE report in the CRF, including date of event, admissions, diagnosis details, date of discharge or death. Those SAEs considered a suspected unexpected serious adverse reaction (SUSAR) will be marked "possible SUSAR" on the SAE report. The investigator will also attach a discharge/medical summary and fax to the CTEU with the SAE report within 24 hours. Any other SAE not fitting the criteria above will be reported on the "other SAE" report in the CRF.

The CTEU will review all serious adverse event/reaction reports before sending an electronic copy to the SAEC for adjudication. All reported SAE/Rs will be adjudicated by the SAEC to assess the seriousness and causality between the IMP and/or concomitant therapy and serious adverse event/reaction. This assessment must take place within two working days upon receipt of the electronic copy of the form. If the SAEC judge the SAE/R to constitute a SUSAR, the CTEU will ensure expedited reporting procedures are followed.

#### Suspected unexpected serious adverse reactions (SUSARS)

A SAR can be considered unexpected when the adverse reaction, the nature or severity of which is not consistent with the applicable product information. All suspected unexpected serious adverse reactions related to an IMP, which occur during the trial, are subject to expedited reporting.

#### Reporting of SUSARs

The CTEU will unblind the SUSAR report before sending it electronically to the SAEC with the medical summary attached. The SAEC will review the report and summary within two working days and adjudicate whether the event constitutes a SUSAR. The SAEC will inform the CTEU of their decision to ensure expedited reporting procedures are followed if necessary.

#### Expedited reporting of SUSARs

All SUSARs will be reported to the MHRA and NHS REC, by the trial co-ordinating site (CTEU). All SUSAR reports will be unblinded by the CTEU prior to submission. A SUSAR, which is fatal, or life threatening will be reported to the MHRA and the main REC by the CTEU as soon as possible and within 7 days after the CTEU become aware of the event. A SUSAR, which is not fatal, or life threatening must be reported to the MHRA and main Research Ethics Committee (REC) as soon as possible and within15 days after the CTEU become aware of the event. The CTEU will inform all trial investigators of all reported SUSARs within 15 working days.

#### Annual reporting

The CTEU will submit annual safety reports of all suspected serious adverse reactions in accordance with regulatory requirements to the MHRA and Main REC. Annual safety reports will be submitted to the MHRA on the date of the original clinical trials authorisation. Annual progress reports will also be submitted to the main REC on the same date. Annual safety reports will also be submitted to the Data Monitoring Committee for review.

#### Participant withdrawal

Investigators must ascertain the reasons for the withdrawal due to adverse events, failure to attend, non-compliance, withdrawal of consent or other reasons. The withdrawal form must be faxed to the CTEU within five working days, unless withdrawal is due to a SAE, in which case the investigator will follow SAE reporting procedures.

#### Unblinding

The treatment code must not be broken except in medical emergencies when the appropriate management of the patient necessitates knowledge of the treatment. Each pharmacy will keep a copy of the randomisation allocation list, in case unblinding is necessary. Should a SUSAR occur, the CTEU will unblind the patient and follow expedited reporting procedures.

#### Indemnity

Patients that are enrolled into the study are covered by indemnity for negligent harm through the standard NHS Indemnity arrangements. The University of Sheffield has insurance to cover for non-negligent harm associated with the protocol. IL1-ra is commercially available for the treatment of inflammatory arthritic conditions. The rate of serious side effects attributable to treatment is <1%. This will include cover for additional health care, compensation or damages whether awarded voluntarily by the Sponsor, or by claims pursued through the courts. Incidences judged to arise from negligence (including those due to major protocol violations) will not be covered by study insurance policies. The liability of the manufacturer of IL1RA (Amgen Corporation) is strictly limited to those claims arising from faulty manufacturing of the commercial product and not to any aspects of the conduct of the study.

#### Ethics

This study will conform with the Declaration of Helsinki guidelines on research involving human subjects. The study protocol will be submitted to the Multi-centre Research Ethics Review Committee, and Ethics Committees at each centre participating in the study and approval obtained before the study commences.

#### Publication policy and dissemination of results

The results from the trial will be submitted for publication in a major journal irrespective of the outcome. The Trial Steering Committee will be responsible for approval of all manuscripts arising from the study prior to submission for publication. Sub-studies of centre-specific data may only be carried out with the knowledge and approval of the Trial Steering Committee.

Any individual authorship of presentations and reports related to the study will be on behalf of the collaborative group. The final follow-up study results paper will name local co-ordinators as well as those involved in central co-ordination and trial management.

At the end of the study, patients will be able to request a copy of the results of the study from the investigator at that site.

#### Proposed study timetable

March 2006 – Grant awarded by MRC

Jan 2007 – Recruitment starts

Dec 2009 – Recruitment completed

Jan 2010 – Last patient completes 30 day follow up

2010 – Publication of data

## Abbreviations

CRP – C Reactive Protein. IL-1 – Interleukin 1. IL-1ra – Interleukin 1 receptor antagonist. ACS – Acute Coronary Syndrome. ECG – Electrocardiogram. AUC – Area under the curve. REC – Research Ethics Committee. IMP – Investigational Medicinal Product.

## Competing interests

Professor David Crossman declares holding limited stock in "IL-Genetica". There would be no direct benefit to this company from the trial.

All other co-author(s) declare that they have no competing interests.

## Authors' contributions

DC: Conceived of the study, participated in the design and co-ordination and helped to draft the manuscript. AM: Participated in the design and co-ordination and helped to draft the manuscript. JG: Participated in the design and co-ordination and helped to draft the manuscript. JGr: Participated in the design and co-ordination and helped to draft the manuscript. AH: Participated in the design and co-ordination and helped to draft the manuscript. KF: Participated in the design and co-ordination and helped to draft the manuscript. AL: Participated in the design and co-ordination and helped to draft the manuscript. MF: Participated in the design and helped to draft the manuscript. BL: Participated in the design and co-ordination and helped to draft the manuscript. CF: Participated in the design and co-ordination and helped to draft the manuscript.

All authors read and approved the final manuscript. Each author is supported directly by their host institution.

## Appendices

### Appendix 1 – Pharmacy locations

#### Sheffield Teaching Hospitals NHS Trust

Dr David Smith

Pharmacy Information Services Manager

Northern General Hospital

Herries Road

Sheffield

S5 7AU

#### Edinburgh Royal Infirmary

Prof Norman Lannigan

Head of Pharmacy

Royal Infirmary of Edinburgh

51 Little France Crescent

Edinburgh

EH16 4SA

#### Leeds General Infirmary

Dr Caroline Bedford

Clinical Trials and Unlicensed Medicines Manager

Leeds Teaching Hospitals NHS Trust

Leeds

#### Labelling

Examples of the labelling can be found appended to the Summary of Product Characteristics.

#### Storage

Store in a refrigerator (2°C – 8°C).

#### Manufacturer's authorisation

EU/1/02/203/001-003

#### Transportation of samples to core lab

Samples will be stored in -20°C freezers at each centre. All participating centres have research freezers available for use. FedEx will then send samples in batches to the core labs.

## Appendix 2 – Sub study protocols

### 1. Cardiac Magnetic Resonance imaging sub study

The Leeds cohort of patients will undergo baseline and follow-up Gadolinium enhanced CMR as a quantitative measurement of extent of myocardial injury. We wish to evaluate the possible mechanisms of any potential benefit and, therefore, feel that investigation of myocardial function, infarct size and oedema is justified. CMR is an accurate and reproducible technique for making these assessments. CMR can distinguish between scar tissue (following myocardial infarction/acute coronary syndromes) and normal (viable) myocardium using the late gadolinium hyper-enhancement technique [38,39]. CMR can also differentiate acute from chronic infarction, based on the principle that acute infarctions are surrounded by tissue oedema secondary to the inflammatory process [40]. Thus, CMR is well suited to extend our understanding of the mechanism of any potential benefit from IL-1ra treatment at the time of NSTEMI ACS. Therefore, we plan a CMR sub-study in the Leeds patients who will have a CMR scan within 48 h of presentation using the protocol outlined below. A follow-up scan, using the same protocol, will be performed after the completion of randomised therapy. Quantification of myocardial oedema, myocardial scar and ventricular volumes/function will be performed on both scans. Exploratory statistical analysis will be undertaken for serial change in these measures within patients and between groups.

#### CMR imaging procedure

1) Localisers and breath-hold plan scans to define LV short axis. 2) Resting wall-motion sequence. Multiphase HLA, VLA and short axis cine stack (10–12 slices) using a fast gradient echo sequence to assess wall motion. Then in identical scan planes; Breath hold, black blood, T2-weighted triple inversion recovery sequence. 3) Late gadolinium hyper-enhancement CMR: T1-weighted, segmented inversion-recovery sequence 15 minutes after intravenous injection of Gadolinium (0.2 mmol/kg). This protocol will be piloted on 20 patients before the start of the main clinical trial.

### 2. ST-Holter segment analysis sub study

As soon as possible after recruitment, between days 2 and 4 of treatment, Sheffield patients will be identified as suitable for ST segment monitoring. Exclusion criteria will include bundle branch block, significant conduction abnormality, digoxin therapy or serious electrolyte disturbance. The presence of other potentially confounding features, such as mitral valve prolapse, posture change, changes due to body habitus and temperature change will be assumed to be similar in the two groups. A Holter monitor will be attached using the 3 standard leads for 24 h. If catheterisation be performed during the period of the recording, an appropriate entry will be made on the time log prior to the monitor being removed and replaced afterwards. If revascularisation be undertaken, the monitor will not be replaced. The monitor will be removed by one of the ECG technicians after 24 h (or as just stated) and analysed by Ms Gomersall (an experienced ECG technician who works in Sheffield), using the standard Reynolds protocol for ST segment analysis. She will be blinded to the identity of the treatment. The number of ischemic events, their frequency and total duration will be compared in both treatment groups.

### 3. Forearm endothelial cell response sub study

This study will examine the effect of Interleukin-1 receptor antagonist (IL-1ra) on vasomotor and fibrinolytic function in the forearm circulation of patients with acute coronary syndromes. The pro-inflammatory cytokines Interleukin-1 (IL-1) and tumour necrosis alpha-α (TNF-α) have been implicated in the initiation and amplification of local and systemic inflammatory responses leading to the development of, and progression of atherosclerosis. However the direct *in vivo *effects of cytokines in patients with IHD on endothelial vasomotor and fibrinolytic function are unknown. As acute coronary syndromes are associated with both impaired fibrinolytic function and transient elevation of acute phase proteins we wish to establish whether IL-1ra affects endothelial function.

Patients will be recruited for the main study. Those recruited in Edinburgh (n = 62) will be asked if they are also prepared to participate in this sub-study. Recruited subjects will attend on two occasions. A comparison of IL-1ra versus placebo on endothelial function will be undertaken. The first study will be performed on days 2–4 following recruitment, at a standardised time following administration of either IL-1ra or placebo (typically 1–4 h). The second study will be performed on day 14 at the same time of day as the first study. Volunteers will have fasted and avoided caffeine for at least four hours and avoided alcohol for 24 hours prior to each study. The subjects will undergo brachial artery cannulation with a 27 standard wire gauge under controlled conditions. Forearm blood flow will be measured in the infused and non-infused arms by venous occlusion plethysmography using mercury-in-silastic strain gauges. Blood samples will be obtained from 17-gauge venous cannulae inserted into the antecubital fossae of both forearms.

Selective infusions into the brachial artery of the non-dominant arm will be undertaken. Substance P will be infused at 2, 4, 8 pmol/min, acetylcholine at 5, 10 and 20 μg/min (endothelium-dependent vasodilator that does not release t-PA), and sodium nitroprusside at 2, 4 and 8 μg/min (endothelium-independent vasodilator that does not release t-PA) will be infused for 5 min at each dose. Blood samples will be taken simultaneously from each forearm at baseline, immediately following, and at 4 further time points after the TNF-α infusion (total less than 200 mls on each occasion). These time points have been derived using the results of previous work to minimise non-contributory venous samples. Blood will be taken to assess stimulated coagulation factors and cytokines. Samples for plasma cotinine, haematocrit, white cell count will also be taken.
